# Genetic Engineering of Lysogenic–Lytic Switch Genes Improves *Burkholderia* Phage Killing Efficacy

**DOI:** 10.3390/ijms27062772

**Published:** 2026-03-18

**Authors:** Pacharapong Khrongsee, Sarah M. Doore, Nawarat Somprasong, Herbert P. Schweizer, Yu-Ping Xiao, Kuttichantran Subramaniam, Ayalew Mergia, Apichai Tuanyok

**Affiliations:** 1 Department of Infectious Diseases and Immunology, College of Veterinary Medicine, University of Florida, Gainesville, FL 32608, USAmergiaa@ufl.edu (A.M.); 2 Faculty of Veterinary Science, Prince of Songkhla University, Songkhla 90110, Thailand; 3 Department of Microbiology and Cell Science, University of Florida, Gainesville, FL 32611, USA; sdoore@ufl.edu; 4 Department of Biological Sciences, The Pathogen and Microbiome Institute, Northern Arizona University, 1395 S. Knoles Dr., Flagstaff, AZ 86001, USA; 5 Emerging Pathogens Institute, University of Florida, 2055 Mowry Rd., Gainesville, FL 32610, USA

**Keywords:** *Burkholderia pseudomallei*, melioidosis, prophage, phage engineering

## Abstract

*Burkholderia pseudomallei*, the causative agent of melioidosis, presents significant challenges in both treatment and environmental decontamination. Bacteriophages, or phages, are increasingly being explored as potential diagnostic, therapeutic, and biocontrol agents against this bacterial pathogen. Our recent investigation has shown that most *B. pseudomallei* genomes contained prophage(s) associated with specific tRNA gene loci, prompting us to explore these detectable prophages as sources of temperate phages for further applications. Transcriptomic profiling of *B. pseudomallei* Bp82, a model strain that possesses three different prophages, revealed high expression levels of the integrase and certain transcriptional regulatory genes within its prophages during normal exponential growth. Using one of its temperate phages, namely φBP82.2, a P2-like phage, as a model, we investigated the lysogenic–lytic control mechanisms. Mutagenesis of the integrase gene, *phiBP82.2_gp51*, did not improve killing activity compared to the wildtype phage. In contrast, deletion of *phiBP82.2_gp38*, a putative transcriptional regulatory gene, and two downstream hypothetical protein genes, *phiBP82.2_gp36* and *phiBP82.2_gp37*, resulted in significant lytic improvement. We conclude that these genes play a crucial role in the lysogenic–lytic switch of φBP82.2, suggesting a new avenue for engineering temperate phages for future applications.

## 1. Introduction

*Burkholderia pseudomallei* is a Gram-negative saprophytic bacterium found in soil and fresh water [1,2,3]. It poses a significant challenge to tropical and sub-tropical regions worldwide, serving as the causative agent of melioidosis in both humans and animals [4,5,6,7]. Melioidosis is considered a difficult-to-treat disease due to the bacterium’s natural multidrug resistance and its ability to survive intracellularly [8]. Thus, effective antibiotic treatments for melioidosis necessitate a prolonged course, often extending up to 20 weeks and incurring high costs. A select few antibiotics have demonstrated efficacy against *B. pseudomallei*, involving an acute phase with parenteral ceftazidime, amoxicillin–clavulanic acid, or meropenem, followed by an eradication phase with oral trimethoprim–sulfamethoxazole [9,10,11,12]. While these antibiotics have proven effective against *B. pseudomallei*, the prolonged treatment duration raises concerns about antibiotic resistance [8,13,14]. Moreover, the widespread presence of *B. pseudomallei* in the environment also complicates efforts to decontaminate affected areas [15]. These dual problems complicate efforts to address melioidosis in many countries, raising serious public health concerns.

In response to these challenges, researchers are currently exploring bacteriophage (phage) therapy as a promising alternative, offering potential for diagnostic, therapeutic, and environmental biocontrol applications [16,17,18,19,20]. However, phages are highly host-specific, and environmental bacteria commonly develop resistance to phages. Finding the right phage(s) to target most *B. pseudomallei* strains may be difficult, which hinders the progress of phage research for this bacterium. Although phages infecting *B. pseudomallei* were first reported as early as 1956 [21], only a few lytic phages, such as ΦST79 and ΦvB_BpP_HN01, have been recently characterized [17,19]. The GenBank database indicates that the majority of known *B. pseudomallei* phages are temperate and are associated with the prophage regions of the bacterial genomes, where the phage persists in the bacterial chromosome after its DNA enters the bacterium [22]. Temperate phages usually contain a site-specific recombination (SSR) sequence, also known as a phage attachment site (*attP*), in their genomes. This specific DNA sequence is used to recombine its genome with a specific homologous sequence on the bacterial chromosome known as the *attB* site [23]. Following integration, a prophage or prophage island is formed and becomes flanked by hybrid attachment sites known as *attL* and *attR*, which result from the combination of the *attP* and the *attB* sites. The prophage is also replicated when the host cell divides and is believed to play an important role in microbial evolution [22,23,24].

Our group has reported that most prophage regions are associated with genomic islands in *B. pseudomallei*, where the foreign genetic materials recombine into the bacterial chromosome, which may allow the bacteria to survive in various environmental conditions [24]. We also observed that the SSR events frequently occurred in highly conserved regions of the bacterial chromosome, such as at genes that encode tRNAs, where prophages are located downstream of the genes flanked by the SSR sequences. We also observed that most *B. pseudomallei* strains contain at least one lysogenic phage [22]. Based on these observations, we used bioinformatic tools to identify the prophage regions of multiple *B. pseudomallei* strains. This was done by tracking a short direct repeat sequence of tRNA genes in *B. pseudomallei* genomes obtained from GenBank. This strategy allowed us to easily predict intact prophages and the potential temperate phages that infect *B. pseudomallei*. However, the success in isolating these temperate phages from *B. pseudomallei* raised an important question of whether we could utilize these phages to infect other *B. pseudomallei* strains. We have learned that their killing efficacy is relatively poor, largely due to their temperate nature. Thus, understanding the regulatory mechanisms governing the lysogenic life cycle of these phages is essential. Such knowledge may allow us to engineer these phages to more effectively kill *B. pseudomallei*, making them more suitable for downstream applications. These temperate phages could then serve as a substantial resource for future applications if their lysogenic to lytic phenotypes can be specifically modified.

In brief, this investigation was developed based on the observation that certain prophage genes, such as phage integrase and lysogeny regulatory genes, were always expressed during the exponential growth phase, while their structural protein genes were mostly silent during the normal growth of *B. pseudomallei*. This led to the discovery of the lysogenic–lytic control mechanisms in a prophage known to be associated with phage φBP82.2, a P2-like phage in *B. pseudomallei* Bp82.

## 2. Results

### 2.1. Genomic Analysis of B. pseudomallei Prophages

**Figure 1:**
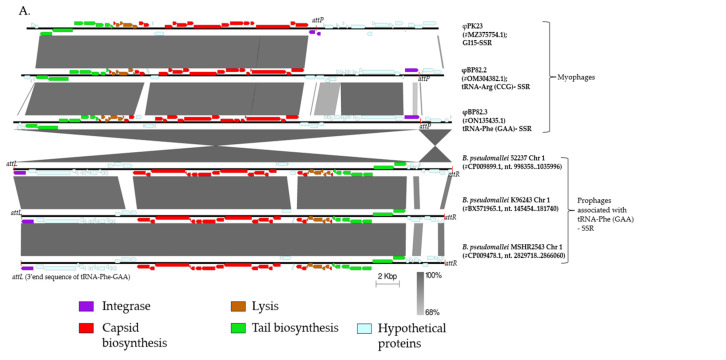

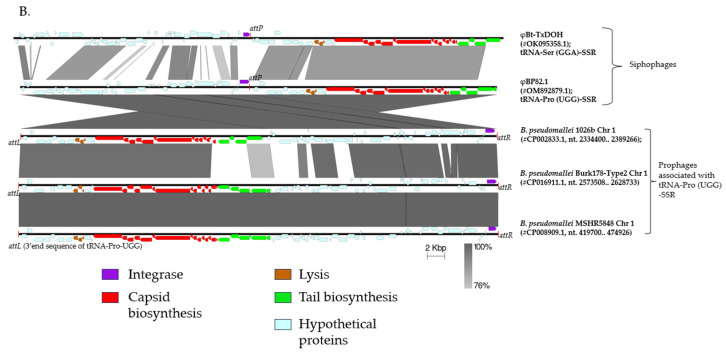
Genomic comparison of temperate phage genomes or prophage DNA sequences associated with *B. pseudomallei* and *B. thailandensis*. Phage structural and late genes are indicated by color-coded arrows (red, brown, and green) corresponding to structural modules, while integrase genes are indicated in purple. (**A**) Myophages, φPK23, φBP82.2, and φBP82.3, are known to be associated with genomic island 15 (GI15), the tRNA-Arginine (anticodon CCG) gene, and the tRNA-Phenylalanine (anticodon GAA) gene, respectively. (**B**) Siphophages, φBt-TXDOH and φBP82.1, are known to be associated with the tRNA-Serine (anticodon GGA) gene and the tRNA-Proline (anticodon UGG) gene, respectively. Phages or prophages sharing the same site-specific recombination (SSR) sites exhibit highly similar gene content across their genomes.

We previously observed that most prophages in *B. pseudomallei* are associated with at least eight tRNA gene locations. These prophages serve as sources for two major temperate phage groups, myophage-like and siphophage-like in morphology and genome organization [22]. In addition, comparative genomic analysis of available *Burkholderia* myophages genomes reveals a high degree of conservation across species. These phages are classified as P2-like phages and belong to the *Peduoviridae* family, which includes the well-characterized Enterobacteria phage P2. The structural genes encoding capsid, tail, and lysis proteins are highly conserved within this group. However, variability was observed in the enzymatic gene regions (comprised of non-structural genes), particularly within one-third of the phage genome if these phages had different integration sites, e.g., different tRNA genes, or different genomic islands (GIs). As shown in Figure 1A, φBP82.2 was associated with tRNA-Arginine (anticodon CCG) and φBP82.3 with tRNA-Phenylalanine (anticodon GAA), while φPK23 associated with a prophage within genomic island 15 (GI 15), a non-tRNA gene location (Figure 1A; [22,25]). In contrast, *Burkholderia* siphophages exhibit greater diversity and possess genomes approximately 1.5 times larger than those of *Burkholderia* myophages. Comparative analysis of prophage regions reveals some structural gene variations among siphophages when located at different genomic sites; for example, φBt-TXDOH, which is associated with the tRNA-Serine (anticodon GGA) gene on chromosome 2 of *B. thailandensis* TXDOH, versus φBP82.1, associated with the tRNA-Proline (anticodon UGG) gene on chromosome 1 of *B. pseudomallei* Bp82 (Figure 1B). Nevertheless, when siphophages are integrated at the same tRNA gene locus, their genomes remain conserved, similar to the pattern observed in myophages. In accordance with the current taxonomy of the International Committee on Taxonomy of Viruses (ICTV), the myophage-like prophages represented by φBP82.2 and φBP82.3 are classified as P2-like peduoviruses within the family *Peduoviridae*, class *Caudoviricetes*, similar to the previously described *B. pseudomallei*-specific phage φPK23 [25]. In contrast, φBP82.1 is referred to here descriptively as a siphophage-like temperate phage pending deeper taxonomic assignment.

Another intriguing finding from our prophage genomic analysis is the strong correlation between phage-encoded integrase genes and tRNA-SSR sequences. For prophage regions linked to a particular tRNA-SSR site, such as prophages integrated at the tRNA-Phe locus in *B. pseudomallei* strains 52237, K96243, and MSHR2543 (Figure 1A), or at the tRNA-Pro locus in strains 1026b, Burk178-Type2, and MSHR5848 (Figure 1B), the corresponding phages consistently encode the same integrase gene sequence. In addition, the non-structural protein-coding regions, which typically comprise approximately one-third of the phage genome, are largely conserved among prophages integrated at the same SSR locus. In addition, a comparison of intact phage genomes with their integrated prophage counterparts, such as φBP82.3 versus prophages at the tRNA-Phe gene locus, and φBP82.1 relative to prophages at the tRNA-Pro, reveals that the phage genomes are split at the SSR junction upon integration. This genomic rearrangement results in the repositioning of the integrase gene to either the first or last open reading frame within the prophage region following chromosomal integration. The prophage boundaries analyzed in this study are consistent with the prophage regions previously validated using PHASTER in our earlier publication [22].

### 2.2. Isolation of a Lysogenic Clone of B. pseudomallei 576mn Following φBP82.2 Infection

**Figure 2:**
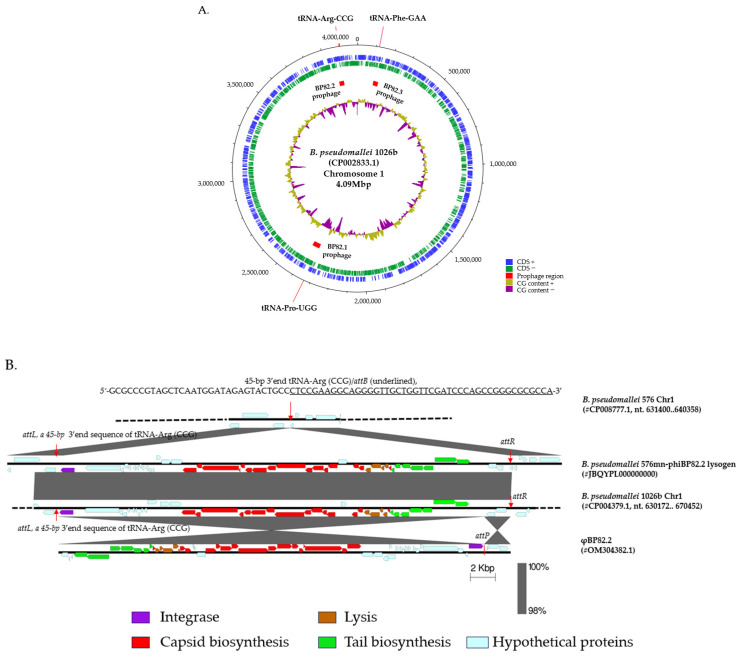
Three prophages in the *B. pseudomallei* 1026b genome, and the integration of a model phage φBP82.2 from its attenuated derivative strain Bp82 into the chromosome of *B. pseudomallei* 576mn, a susceptible host strain: (**A**) the genomic locations of these prophages on chromosome 1 of *B. pseudomallei* 1026b, and (**B**) the prophage formation in *B. pseudomallei* 576mn following φBP82.2 infection. In panel B, the genomic comparison showed that the φBP82.2 genome was integrated into the 3′end sequence of the tRNA-Arg (anticodon CCG) gene through site-specific recombination. The resulting prophage in 576mn was flanked by a short 45-bp repeat, identical to the 3′end sequence of tRNA-Arg gene at both ends, termed *attL* and *attR*. This prophage in 576mn is identical to the prophage associated with the tRNA-Arg gene on chromosome 1 of *B. pseudomallei* 1026b.

To investigate whether the temperate phages exhibit specificity for a particular phage integration site, known as the *attB* sequence of the bacterial genome, we used a temperate phage φBP82.2 from *B. pseudomallei* Bp82, an attenuated derivative of *B. pseudomallei* 1026b, to infect its susceptible host *B. pseudomallei* 576mn, as previously described [22]. Bp82 carries three functional prophages on chromosome 1 (Figure 2A [22]). Following infection, a lysogen was isolated from a resulting bacterial colony grown inside the plaque of φBP82.2 and subjected to whole-genome sequencing. The analysis showed that φBP82.2 integrated into the tRNA-Arg (CCG) gene of strain 576mn, precisely at the same site as in the Bp82 genome (Figure 2B). We noted that the phage integration *attB* site corresponds to a 45-bp 3′end sequence of the tRNA-Arg gene. As observed previously, the phage genome containing an *attP* sequence was recombined with this *attB* sequence through homologous recombination, resulting in the integrase gene being repositioned as the first gene in the integrated prophage downstream of the tRNA-Arg gene. Furthermore, the prophage region was flanked by an identical sequence *attP/attB* (also known as *attL* and *attR*) right at the end of the prophage. Since the integrase gene is located next to the *attB* site, it could play a major role in mediating site-specific recombination at the *attB* site during phage integration, and a reverse recombination for phage excision to enter the lytic cycle. Genomes of *B. pseudomallei* 576mn and its lysogenic derivative strain 576mn-phiBP82.2 are available through GenBank with accession numbers: JBQYPK000000000 and JBQYPL000000000, respectively.

### 2.3. Expression Profile of the Prophages in B. pseudomallei Bp82 During Normal Exponential Growth

**Figure 3:**
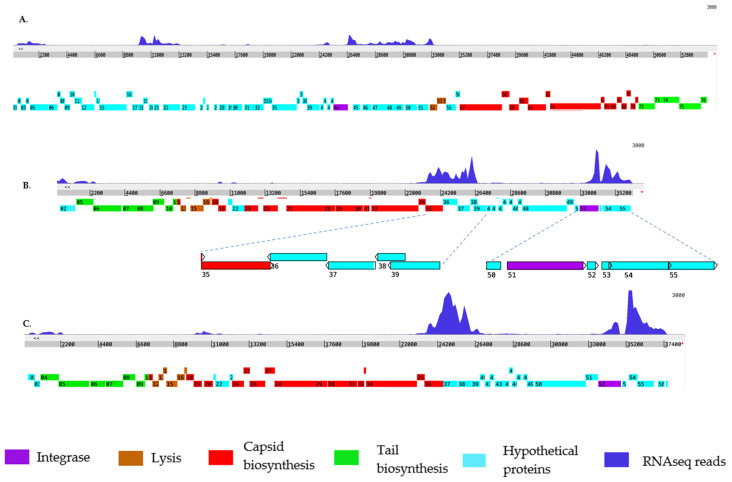
Mapping of RNA-seq reads against three temperate phage genomes of *B. pseudomallei* Bp82 to simplify the visualization of the expression of prophage genes. Normalized RNA-seq read coverages (blue) were mapped to each temperate phage: (**A**) φBP82.1, (**B**) φBP82.2, and (**C**) φBP82.3. Overall, most of the structural protein genes (color-coded in red, green, and brown) of all three prophages were silent, while integrase genes (purple) and some hypothetical protein genes (cyan) were highly expressed in prophages associated with both myophages φBP82.2 and φBP82.3. We noted that the integrase gene in the φBP82.1 prophage was not expressed. Genes *gp36–38* in φBP82.2, predicted as a lysogeny regulatory gene cassette, were also highly expressed.

To identify prophage genes involved in the bacterial lysogenic life cycle, we conducted RNAseq analysis during the normal exponential growth of *B. pseudomallei* Bp82. Expression profiles of the prophage genes were generated by mapping normalized RNA-seq read coverage to each phage genome for a simplified visualization (Figure 3). Applying a 3000 read coverage cut-off window view, we observed that both prophages sourced from the myophages, φBP82.2 and φBP82.3, displayed higher read coverages compared to the siphophage, φBP82.1. This finding correlates with the higher plaque-forming activity of φBP82.2 and φBP82.3 than that of φBP82.1 on the *B. pseudomallei* 576mn host. Furthermore, structural protein genes encoding tail, capsid, and endolysin genes were transcriptionally silent across all three phages, indicating a dormant state consistent with lysogeny. Both myophages exhibited nearly identical gene expression profiles within the non-structural part of their genome (Figure 3B,C). This similarity persisted despite gene variations due to the different tRNA-SSR location of each prophage. Moreover, both prophages associated with φBP82.2 and φBP82.3 showed a consistent pattern of gene expression, with high expression of the integrase gene. Additionally, a cluster of lysogeny regulatory genes upstream of the integrase also exhibited high expression in both prophages. Furthermore, a set of hypothetical genes downstream of the integrase, with unknown functions, displayed high expression. In contrast, the integrase gene in the prophage associated with φBP82.1 was not expressed, while genes *gp45–52*, predicted to act as repressors, were expressed (Figure 3A).

### 2.4. Deletion of the Integrase Gene gp51 in the φBP82.2 Prophage Resulted in the Loss of Phage Excision

**Figure 4:**
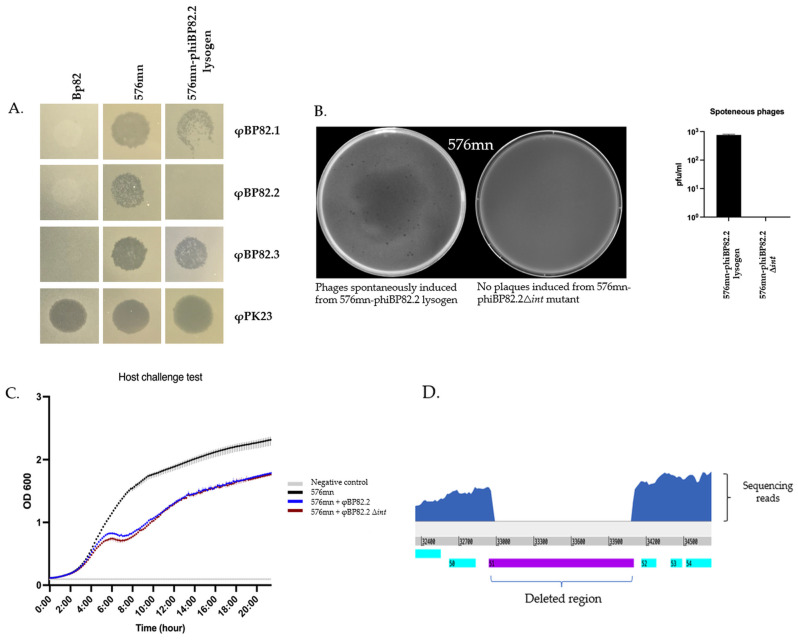
Phenotypic characterization of *B. pseudomallei* 576mn-phiBP82.2 lysogen resulting from φBP82.2 integration: (**A**) spot test results of φBP82.1, φBP82.2, φBP82.3, and φPK23 (control) against *B. pseudomallei* strains Bp82 (source strain), 576mn (a susceptible host), and 576mn-phiBP82.2 lysogen; (**B**) plaques observed from 576mn-phiBP82.2 lysogen on 576mn lawn following spontaneous induction, while no plaques observed from its integrase gene (*gp51*, *int*) deleted mutant; (**C**) killing efficacy of the φBP82.2Δ*int* mutant (resulted from *int*-trans complementation), against 576mn with no improvement when compared with the wildtype phage φBP82.2. The experiment was performed in triplicate. (**D**) Whole-genome sequencing of the φBP82.2Δ*int* confirmed the absence of its integrase gene.

To investigate the role of an integrase gene in φBP82.2 prophage, we used the lysogenic *B. pseudomallei* 576mn-φBP82.2 as a model strain. This is because *B. pseudomallei* 576mn does not contain any intact prophages in its genome, which eliminates potential confounding factors of integrase genes from other prophages. We observed that this lysogenic strain exhibited immunity against φBP82.2 infection, while it was susceptible to φBP82.1 and φBP82.3 (Figure 4A).

To assess the function of the integrase gene in φBP82.2 prophage, we engineered a suicide plasmid vector, pExKm5 [26], to target the integrase gene (*int*, *gp51*) present in the integrated φBP82.2 prophage in the lysogenic *B. pseudomallei* 576mn-phiBP82.2. Upon transforming the plasmid into the bacteria, we successfully isolated merodiploid colonies and counterselection, resulting in a marker-less mutagenesis of the *gp51* gene. The resulting integrase deletion mutant was confirmed by PCR, designated as *B. pseudomallei* 576mn-phiBP82.2Δ*int*. Sequencing of the mutant phage, φBP82.2Δ*int*, confirmed the deletion of the integrase gene (Figure 4D and GenBank accession no. PX400618). We did not observe any phage released from this mutant when it was spontaneously induced in liquid culture and propagated with the wildtype 576mn (Figure 4B). To test if this was due to the deletion of the phage integrase gene, we then conducted a trans-complementation of the integrase gene controlled by the *lac* promoter on a recombinant plasmid pBIC (pBRR1K::*phiBP82.2_gp51*) into the mutant strain. After six hours of IPTG induction, the supernatant was collected for spontaneous phage analysis. As expected, we successfully observed plaque formations on the double agar overlay culture of 576mn. The phage produced from the 576mn-phiBP82.2Δ*int* mutant with *int*-trans complementation was used in the phage-host challenge test for the comparison of the killing efficacy with the wildtype φBP82.2. However, the killing efficacy of this mutant phage did not improve compared to the wildtype φBP82.2 (Figure 4C). This suggests that the integrase does not play a major role in the switch to the phage lytic life cycle.

### 2.5. Lysogeny Regulatory Gene Involvement in the Lysogenic–Lytic Switch

To further identify the genes responsible for maintaining lysogeny, we hypothesized that these genes would be highly expressed during the normal growth of bacteria to suppress the excision of phages. Based on the RNAseq data, we observed a group of highly expressed genes corresponding to *gp36*, *gp37*, and *gp38* in the φBP82.2 prophage, which were located near the integrase gene. Although prophage φBP82.3 contains a different set of genes at the same genomic location when aligned with φBP82.2, genes in this location were also highly expressed despite limited sequence similarity. To investigate the function of these genes in the model φBP82.2 prophage, we proceeded with the mutagenesis of the *gp36*, *gp37*, and *gp38* genes in the lysogenic *B. pseudomallei* 576mn-phiBP82.2 using the same approach described above. We successfully resolved the merodiploid following counterselection of the *gp36* deletion mutant by plating on medium containing 15% sucrose. In contrast, no bacterial colonies were recovered after counterselection of the *gp37* or *gp38* deletion mutants. We therefore hypothesized that deletion of these two genes could trigger the activation of the lytic life cycle, resulting in host cell lethality.

**Figure 5:**
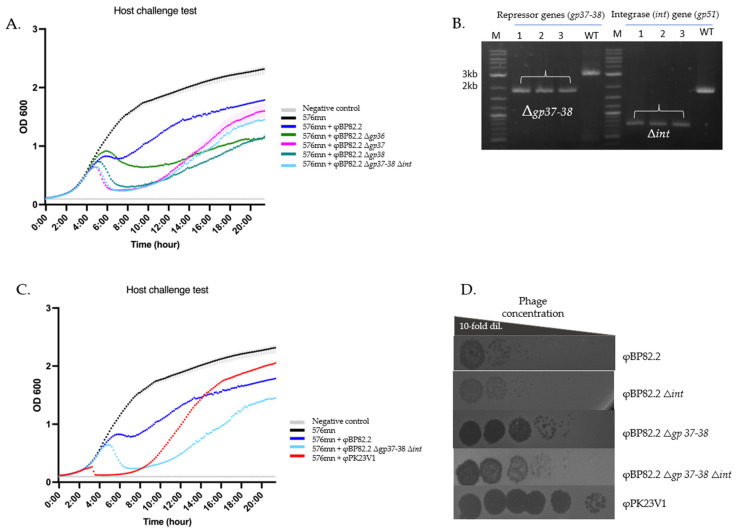
Enhanced killing activity of mutant phages. This figure illustrates the phenotypic changes associated with lysogeny regulatory gene mutant phages. (**A**) Killing curve of the mutant phages, demonstrating that the deletion of *gp36*, *gp37*, and *gp38* genes in φBp82.2 effectively enhanced phage lytic activity. We also evaluated a triple mutant, φBp82.2Δ*gp37–38*Δ*int*, which showed a similar killing activity compared with the φBp82.2Δ*gp37* mutant. (**B**) PCR confirming deletion of the hypothetical lysogeny regulatory genes *gp37–38* and the integrase gene *gp51*. (**C**) Comparison of the killing activity of the φBp82.2Δ*gp37–38*Δ*int* mutant and φPK23V1, a highly *B. pseudomallei*-specific lytic-like phage. The mutant phage exhibited slower killing but better control of bacterial growth compared with φPK23V1. (**D**) Spot test resulting from 10-fold serial dilution of phage preparations. Phage stocks were serially diluted 10-fold and 5 μL of each dilution was spotted onto bacterial lawns to estimate relative phage titers. Deletion of the integrase gene did not result in increased phage production, as indicated by similar clearing patterns across dilutions. In contrast, the *gp37–38* gene-deleted mutant phages showed an approximately 2-log increase in their titers compared with the wildtype phage. Despite this increase, phage titers of the *gp37–38* mutants remained lower than those of the φPK23V1 control.

To assess this, we attempted to resolve the merodiploid by cultivating the suspected clones in LB broth containing 15% sucrose instead of LB agar, with the expectation of recovering mutant phages that could be released into the liquid phase following host cell death. As expected, we successfully recovered the mutant phages φBP82.2Δ*gp37* (*gp37* deletion) and φBP82.2Δ*gp38* (*gp38* deletion). Using the same approach, we successfully created a triple gene deletion mutant, φBP82.2Δ*gp37–38*Δ*int*. The triple gene mutant phage was confirmed by PCR (Figure 5B). A phage-host challenge test of these mutant phages demonstrated a fascinating improvement in the killing efficacy against *B. pseudomallei* 576mn host (Figure 5A). The highest concentrations of the phages also improved by about a 2-log difference (Figure 5D), suggesting that the lysogeny regulatory genes play a crucial role in the lytic switch of these *Burkholderia* P2-like prophages. However, we also performed a comparison of the killing efficacy of the engineered phages with φPK23V1, one of the most *B. pseudomallei*-specific lytic-like phages previously described [25]. The φPK23V1 consistently exhibited superior killing efficacy and higher phage titer compared with the engineered phages (Figure 5C,D).

## 3. Discussion

In the era of advanced genetic modification, the potential of engineered bacteriophages has garnered attention as an alternative agent against antibiotic-resistant bacteria, driven by the intentional design to fix some weaknesses of phages for specific purposes. Two notable examples of successful engineered phages are *Mycobacteriophage* ZoeJ and BPs, which have been effectively utilized to treat multidrug-resistant *Mycobacterium abscessus* in a cystic fibrosis patient [27,28,29]. These temperate phages underwent genetic engineering, involving the deletion of immunity genes and the *attP* site sequence, ultimately transforming the temperate phages into virulent lytic forms. In *B. pseudomallei*, we previously identified a widespread prevalence of prophages in the population, with approximately fifty percent of bacterial genomes containing at least one prophage [22]. Thus, we were curious to explore the possibility of modifying these phages to enhance their lytic activity, aiming to improve the killing efficiency of these phages for downstream applications. Our work has illustrated the process from the initial discovery of the temperate phages and used one of them as a model to engineer for practical use. Here are the main points to discuss:

### 3.1. Temperate Phages Are Common in B. pseudomallei and May Contribute to Host Survival

We have recently demonstrated that the majority of reported bacteriophages infecting *B. pseudomallei* are P2-like myophages belonging to the family Peduoviridae, followed by siphophages. Consistent with this trend, the attenuated strain Bp82 harbors two myophages, φBP82.2 and φBP82.3, and one siphophage, φBP82.1, integrated within its genome, providing a useful model for studying phage biology in this bacterium [22]. One likely advantage of lysogeny is the protection it confers against superinfection by related phages. In support of this, φBP82.2 failed to infect both the 576mn-phiBP82.2 lysogen and the parental Bp82 strain, demonstrating classical superinfection immunity (Figure 4A). These findings suggest that temperate phages may enhance bacterial survival by preventing infection from closely related phages in the environment.

### 3.2. The Integrase Gene Is Required for Phage Excision but Does Not Enhance Lytic Activity

We previously identified the unique phage integration sequences, including site-specific recombination sequences (SSRs) [22]. These elements were conserved when prophages occupied the same genomic loci. In addition, the integrase gene exhibited high expression levels during the exponential bacterial growth in liquid medium, leading us to hypothesize that it may play a crucial role in regulating the dormant prophage stage. To test this hypothesis, we deleted the integrase gene (*gp51*) in the prophage of the lysogenic 576mn-phiBP82.2 strain. The resulting mutant strain was defective in spontaneous phage induction. However, complementation of the integrase gene successfully restored spontaneous induction, leading to the release of the φBP82.2Δ*int* mutant and demonstrating that the integrase gene is required for the phage excision. Despite its essential role in phage excision, deletion of the integrase gene did not enhance phage-mediated killing. This observation suggests that the primary regulatory mechanisms governing the lytic switch are not directly controlled by the integrase. Consistent with our finding, Shitrit and colleagues demonstrated that both the integrase gene and the phage integration site are essential for integration, yet deletion of these genes did not increase the lytic activity of the engineered phage [30]. Together, these results indicate that although integrase function is critical for prophage excision and integration, it does not represent a limiting factor for enhancing lytic activity in this system.

### 3.3. The Deletion of Lysogeny Regulatory Genes Activates the Lysogenic–Lytic Switch

P2-like phages are widely distributed among Gram-negative bacteria, with Enterobacteria phage P2 (GenBank accession no. NC_001895.1) serving as a well-studied prototype. Members of this group are currently classified within the family Peduoviridae and share a modular genome organization consisting of structural, replication, and lysogeny control regions arranged in a conserved orientation. Although sequence similarity among accessory genes can be limited, the overall genome architecture and regulatory organization are often covered across P2-like phages infecting different bacterial hosts.

In our comparative analysis, we performed BLASTp searches (BLAST+ 2.17.0) to assess protein-level similarity between phage P2 and our φBP82.2 phage (Supplementary Figure S1). This analysis revealed limited similarity among structural protein genes, whereas nonstructural or accessory protein genes showed little to no detectable homology. Despite the low sequence conservation, the overall gene orientation was largely conserved across the two genomes, supporting the classification of φBP82.2 within the broader P2-like phage lineage. The highly expressed φBP82.2-associated prophage genes *gp36*, *gp37*, and *gp38*, located on the reverse strand, were transcribed in the opposite orientation relative to genes involved in the lytic cycle. HHpred protein homology suggested that *gp38* contains a predicted helix–turn–helix (HTH) DNA-binding motif, which is commonly found in transcriptional regulators involved in lysogeny control in temperate phage systems. Based on this observation, we hypothesized that these genes may function as regulators of the lytic-lysogenic decision, analogous to the C gene in the P2 phage [31]. Consistent with this hypothesis, deletion of *gp36*, *gp37*, or *gp38* in φBP82.2 successfully triggered activation of the lytic life cycle. A similar phenomenon has been well documented in phage lambda, where deletion of the transcriptional repressor results in obligately lytic phages, underscoring the conserved role of repressor proteins in maintaining lysogeny across diverse temperate phage systems [32].

Supporting this model, Yao and colleagues reported that mutations near promoter regions of similarly oriented reverse-strand genes resulted in strict virulence in *Burkholderia* phage Milagro, a P2-like phage infecting *B. cenocepacia* [33]. The authors proposed that face-to-face promoters (P_L_ and P_R_) in this genomic region compete with one another, leading to antagonistic regulation of lytic genes encoded on the forward strand. A comparable regulatory architecture exists in P2 phage, where the *C* and *cox* genes are arranged in opposite orientations. Binding of the C protein to the *Pe* promoter inhibits *cox* expression, thereby promoting lysogeny. Consistent with this model, transcriptional profiling of φBP82.2 prophage during the exponential growth phase, together with promoter prediction using BPROM (SoftBerry Inc., Mount Kisco, NY, USA), identified a σ70-dependent promoter upstream of *gp38*. In contrast, promoter elements on the forward strand remain poorly defined. Furthermore, the precise inhibitory mechanisms mediated by the *gp37*–*gp38* proteins remain to be elucidated and warrant further investigation.

## 4. Materials and Methods

### 4.1. Bacterial Culture Condition

*Burkholderia* spp. and *Escherichia coli* strains were cultured at 37 °C with aeration in LB media. The attenuated strains *B. pseudomallei* Bp82 [34] and 576mn [35], the biosafe non-select agent strains, were additionally supplemented with 80 mg/L of adenine. Bacteriophages were propagated on bacterial host soft agar by combining 100 μL of log phase 576mn culture with 4.5 mL of molten LB adenine soft agar (0.35% *w*/*v*) in a sterile 15 mL tube. The resulting mixture was poured onto an LB agar plate supplemented with 80 mg/L of adenine. Once the agar solidified, one hundred microliters of the phage solution was dropped onto the bacterial soft agar plate, gently rotated to spread the phage solution over the plate surface, then allowed to dry for 10 min. Following drying, the plate was inverted and incubated at 37 °C overnight, and subsequently, plaque formations were observed. Bacterial growth curves and host challenge tests were conducted by combining 100 mL of fresh 576mn culture at 0.1 optical density 600 nm (O.D. 600), assuming 10^8^ cfu/mL of the bacterial host with 100 mL of 10^5^ pfu/mL phage in 500 mL LB adenine media in a 48-well flat-bottomed plate with a lid. The cultures were shaken using linear shaking mode at 493 cpm in a BioTek Synergy HTX plate reader (Agilent Technologies, Inc., Santa Clara, CA, USA). This process was performed in triplicate at 37 °C. The O.D. 600 nm was measured every 10 min for a duration of 24 h. It is important to note that a low multiplicity of infections (~MOI 0.001) was used due to the limited production of the temperate phages.

### 4.2. Prophage Genome Analysis

We conducted pangenome analysis using the local NCBI BLASTn (v 2.2.18) [36] command line to acquire the coordinate sequences at the locations of the prophages associated with tRNA genes, as detailed in our previous publication [22]. The coordinates encompassing the start and end bases of each prophage region, along with the chromosome strand information, were compiled into a table. This table was then utilized to generate BED files. Subsequently, the BED files were employed to extract a multiFASTA file for each prophage genome using the BEDtools command (v.2.31.0) [37]. Also, the coordinates were used to download the GenBank file of each prophage region from the GenBank database directly. To perform whole phage genome alignments, rigorous comparisons were done in the Artemis Comparison Tool (18.2.0) [38] and Easyfig 2.2.2 OSX [39] to compare the conserved regions of the prophages.

### 4.3. Phage Induction and Lysogen Isolation

The Bp82.2 phage was induced from the *B. pseudomallei* strain Bp82, following the procedure outlined in [22]. The phage solution was spotted on top of the surface of a 576mn LB adenine soft agar plate and incubated overnight. The presence of a clear zone, with colonies growing inside the clear zone, was observed on the next day. A sterile inoculating loop was used to stab the clear zone and was streaked onto a new LB adenine plate, then incubated for 48 h. For the isolation of lysogen colonies, each colony was assigned a number and individually stabbed onto a 576mn LB adenine soft agar plate, followed by overnight incubation. The presence of a clear zone ring around the stabbed colony indicated that it was a lysogen colony. Subsequently, these lysogen colonies were purified three times and confirmed for the *integrase* gene of Bp82.2 through colony PCR.

### 4.4. RNA Sequencing

A 3 mL volume of fresh Bp82 culture with an O.D. 600 of 0.1 was incubated at 37 °C with aeration for 3 h, using triplicate tubes. Subsequently, a 1 mL aliquot of each culture was centrifuged at 16,000× *g* for 1 min to obtain a pellet in each sample. RNA extraction from each bacterial pellet was carried out using the Direct-zol^TM^ RNA Miniprep kit (Zymo Research, Irvine, CA, USA) following the manufacturer’s protocol. For the RNA sequencing method, the samples underwent DNAse treatment with Invitrogen DNAse (RNAse-free, Carlsbad, CA, USA). Library preparation was executed using Illumina’s Stranded Total RNA Prep Ligation with the Ribo-Zero Plus kit and 10-bp unique dual indices (UDI). Sequencing was performed on a NovaSeq X Plus (Illumina, Inc., San Diego, CA, USA), generating paired-end 150 bp reads. Demultiplexing, quality control, and adapter trimming were executed using bcl-convert (v4.1.5). Subsequently, reads were trimmed with Trimmomatic (v0.39) [40] and mapped onto the *B. pseudomallei* 1026b, φBP82.1, φBP82.2, and φBP82.3 genomes using BWA-MEM (v0.7.15-r1140) [41]. The resulting BAM files were visualized on Artemis (v18.2.0) [42]. To allow comparison of transcriptional signals across prophage genomes, read coverage values were normalized to sequencing depth. Normalized read coverage was used to visualize the relative transcriptional activity of prophage genes during lysogenic growth. The RNA-seq analysis presented here was intended to identify highly expressed prophage genes rather than to perform formal differential expression analysis.

### 4.5. Phage Integrase Gene Mutagenesis

A plasmid pExKm5 was provided as a gift by Dr. Herbert P Schweizer at the University of Florida. To mutate the phage integrase (*int*) gene or *phiBP82.2_gp51*, around 900 base pairs upstream and downstream of the gene were amplified using the primers listed in Supplemental Table S1. The PCR fragments were gel-purified using Zymoclean Gel DNA Recovery Kits (Zymo Research Corp., CA, USA). To construct the pExKm5-Bp82.2Δ*int* plasmid, we modified a method from López CM et al., 2009 [26]. Briefly, the pExKm5 was digested by *EcoRI*-HF and *NotI*-HF (New England Biolabs, Ipswich, MA, USA) and gel-purified as the previous PCR products. The digested plasmid and the PCR fragments were assembled using NEBuilder HiFi DNA Assembly Master Mix (New England Biolabs, MA, USA) using the manufacturer’s protocol. The assembled plasmid was then transformed into *E. coli* DH5a-competent cells on an LB plate supplemented with 50 ug/mL of kanamycin (Km) and 5-bromo-4-chloro-3-indolyl-β-d-galactopyranoside (X-Gal). White colonies were selected and used for plasmid amplification. *B. pseudomallei* 576mn-phiBP82.2 lysogen electrocompetent cells were made in-house by washing three times with 300 mM sucrose as described by Choi KH et al. in 2005 [43]. Approximately 100–200 ng of purified plasmid DNA was then electroporated into the 100 µL competent cells using a 0.2 mm cuvette (Bio-Rad, Hercules, CA, USA) at 2500 V 200 Ω. The transformed cells were recovered in LB broth with shaking at 200–250 rpm for two hours. Then the mixture was plated on LB plates containing 250 μg/mL Km and 50 μg/mL of 5-bromo-4-chloro-3-indolyl-β-d-glucuronide (X-Gluc) for 48 h. A blue Km^r^ colony was picked up and the merodiploid was solved by streaking with the presence of 15% sucrose in YT agar supplemented with 0.8 ug/mL adenine for 48 h. White colonies were then tested by PCR to confirm the mutant lysogen using upstream forward and downstream reverse primers.

### 4.6. Construction of Replicative Plasmid for Integrase Supplementation

Plasmid pBbB1k-GFP (addgene#35342) [44] was used as a plasmid backbone to construct pBBR1K-Bp82.2integrase-chromoprotein (pBIC). The 660 bp chromoprotein gene was amplified from plasmid pSB1C3 (tsPurple chromoprotein; addgene#117848) [45], with primers listed in Supplemental Table S1, using Q5 High-Fidelity DNA Polymerase (New England Biolabs, MA, USA) at an annealing temperature of 55 °C for 45 s and extension at 72 °C for 1 min. An *integrase* gene was amplified from the *phiBP82.2_gp51* gene using primers listed in Supplemental Table S1 at an annealing temperature of 60 °C for 45 s and extension at 72 °C for 90 s. The PCR products were gel-purified using Zymoclean Gel DNA Recovery Kits. To construct a pBIC plasmid, the pBbB1k-GFP plasmid was digested with *NdeI* and *BamHI*-HF (New England Biolabs, MA, USA), then assembled with the purified *phiBP82.2_gp51 DNA* and chromoprotein PCR fragments with NEBuilder HiFi DNA Assembly Master Mix (New England Biolabs, MA, USA), The pBIC was then transformed into competent cells of *E. coli* DH5α for amplification. The successful transformants were identified by the appearance of pink-purple colonies. For integrase complementation to induce the mutant phage, the 576mn-phiBP82.2Δ*int* lysogen was transformed with the pBIC plasmid using the electroporation protocol described above. The recovery cells were plated on LB adenine supplemented with 250 µg/mL Km. Plasmid presence in a clone was confirmed by PCR using primers targeting the *phiBP82.2_gp51* integrase gene. To induce the expression of the integrase to promote spontaneous phage production, the confirmed clone was cultured in LB adenine broth containing 250 µg/mL Km until reaching a log phase and then induced with 0.5 M Isopropyl ß-D-1-thiogalactopyranoside (IPTG) (Sigma-Aldrich, Inc., St. Louis, MO, USA) for 6 h. One milliliter of the culture was then centrifuged at 16,000× *g* for 1 min, and the supernatant was then filtered on a 0.2 µm filter membrane. A volume of 100 µL of the supernatant was plated on top of the 576mn LB adenine soft agar plate. The mutant phages were isolated from plaques after overnight incubation.

### 4.7. Phage Lysogeny Regulatory Gene Mutagenesis

To mutate the phage lysogeny regulatory genes, we used the same technique as described above with all the primers listed in Supplemental Table S1. However, resolution of the merodiploid resulted in host cell lethality with no mutant clone observed on the sucrose-containing LB plate. Therefore, the merodiploid was resolved in liquid media as follows. A blue colony was inoculated in 3 mL of LB-adenine-Km250-X-gluc media overnight. The culture was centrifuged at 4000× *g* for 10 min. The supernatant was discarded, and the pellet was resuspended in 3 mL of LB broth. This washing step was repeated three times to remove the residual antibiotic. The pellet was then resuspended in 3 mL of LB–adenine broth containing 15% sucrose and incubated at 37 °C for 4 h. After incubation, 1 mL of the culture was aliquoted and centrifuged for phage isolation as described above, and then the filtered supernatant was used to perform phage isolation.

## 5. Conclusions

Our study established φBP82.2, a temperate phage from *B. pseudomallei* Bp82, as a tractable model for phage engineering. We demonstrated distinct functional roles for the integrase and the hypothetical lysogeny regulatory genes, with the latter acting as key regulators of the lysogenic–lytic switch in this P2-like phage. Deletion of the integrase gene impaired phage excision, whereas deletion of these lysogeny regulatory genes enhanced lytic activity. However, our results also indicate that deletion of a single lysogeny regulatory gene alone is not sufficient to fully convert a temperate phage into a strongly lytic phage, highlighting the complexity of the regulatory mechanisms controlling the lysogenic–lytic switch. These engineered phage derivatives therefore provide a useful experimental model for studying phage regulatory networks and may help guide future efforts to engineer temperate phages for improved antibacterial activity.

## Data Availability

The data presented in this study are openly available in GenBank [https://www.ncbi.nlm.nih.gov] reference number [JBQYPK000000000, JBQYPL000000000].

## Supplementary Materials

The following supporting information can be downloaded at: https://www.mdpi.com/article/10.3390/ijms27062772/s1.
