# Supplementary material for: Genetic Engineering of Lysogenic–Lytic Switch Genes Improves Burkholderia Phage Killing Efficacy
Source: Int J Mol Sci. 2026 Mar 18;27(6):2772. doi: 10.3390/ijms27062772 (PMC13026187; doi:10.3390/ijms27062772)
Supplement: Supplementary file 1 [file ijms-27-02772-s001.zip › Supplemental Figure S1.pdf]

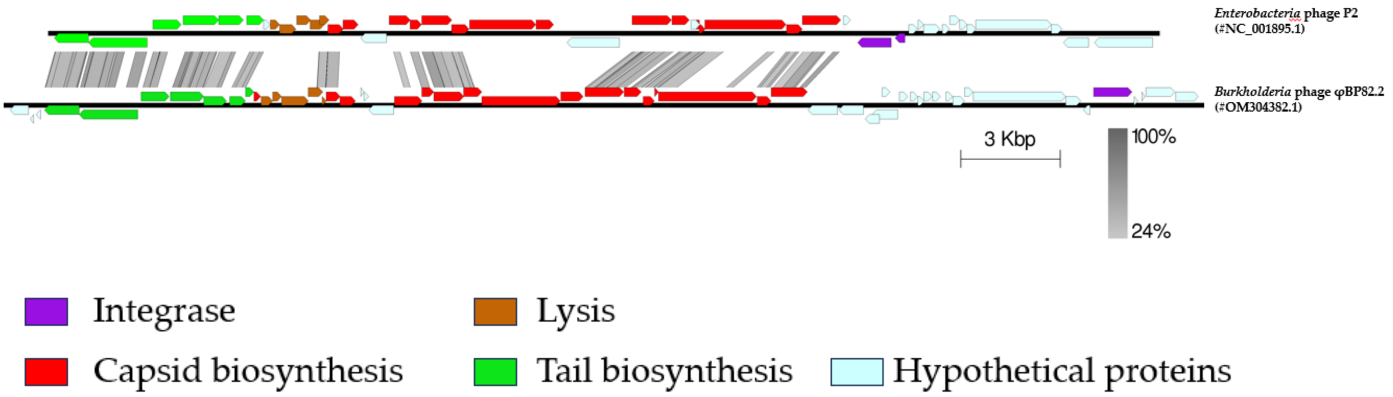

Supplemental Figure S1. Genomic comparison of Enterobacteria phage P2 and *Burkholderia* phage φBP82.2. A comparative genomic analysis of Enterobacteria phage P2 (GenBank accession no. NC_001895.1) and *Burkholderia* phage φBP82.2 (GenBank accession no. OM304382.1) is shown. Structural protein-coding genes are color-coded in red, brown, and green, while the integrase gene is highlighted in purple. Limited similarity was observed among structural genes, whereas no detectable homology was found among nonstructural genes. Despite this low sequence similarity, overall gene orientation is highly conserved between the two phages.
