# Supplementary material for: Genetic Engineering of Lysogenic–Lytic Switch Genes Improves Burkholderia Phage Killing Efficacy
Source: Int J Mol Sci. 2026 Mar 18;27(6):2772. doi: 10.3390/ijms27062772 (PMC13026187; doi:10.3390/ijms27062772)
Supplement: Supplementary file 1 [file ijms-27-02772-s001.zip › Supplemental Table 1.pdf]

Supplemental Table 1. List of plasmids and PCR primers used in this study.

Plasmid constructs	Cloning vector	Cloning sites	Target gene	Primer sequence (5′-3′)
pExKm5-*phiBP82.2△int*	pExKm5	*EcoRI, NotI*	*phiBP82.2_gp51*	Upstream_fw: CCTGTTATCCCTACCCGGGCGCGACAAATGGAACGGCC<br>Upstream_rev: GAAATTTCAGACTTCCTCTTGCTGATCGC<br>Downstream_fw: AAGAGGAAGTCTGAAATTTCACTGAAGTAGG<br>Downstream_rev: GGGATAACAGGGTAATCCCGCATATAGACGACTGCGCG
pExKm5-*phiBP82.2△gp36*	pExKm5	*EcoRI, NotI*	*phiBP82.2_gp36*	Upstream_fw: ATTACCCTGTTATCCCTACCCGGGCACGTGACGGGCCCCTTCACGAT<br>Upstream_rev: GATAGCGCACAACGCTCCCTCGGTGAATTCGTTACAC<br>Downstream_fw: CACCGAGGGAGCGTTGTGCGCTATCTACTGCGGCG<br>Downstream_rev: CTCTAGGGATAACAGGGTAATCCCGCGAAAAACGATCTGGACATTCCGAAG
pExKm5-*phiBP82.2△gp37*	pExKm5	*EcoRI, NotI*	*phiBP82.2_gp37*	Upstream_fw: ATTACCCTGTTATCCCTACCCGGGCGGGAATCGTCCCATCCGAGCGC<br>Upstream_rev: ATTCGTTACACGTGTGCGCATTGCATGTTTTCTTTTTCTCCTGCGGCTGGC<br>Downstream_fw: TGCAATGCGCACACGTGTAACG<br>Downstream_rev: CTCTAGGGATAACAGGGTAATCCCGGTGCCTTCGCAGAAAATCCGCATATC
pExKm5-*phiBP82.2△gp38*	pExKm5	*EcoRI, NotI*	*phiBP82.2_gp38*	Upstream_fw: CCTGTTATCCCTACCCGGGCCCGCAATGGCACGATGCGAG<br>Upstream_rev: TCTCGGTCTCTTGACCACCATTCAAGTTGACAAGTGG<br>Downstream_fw: TGGTGGTCAAGAGACCGAGAAATTAGCGGC<br>Downstream_rev: GGGATAACAGGGTAATCCCGTTTACGAGCGCCTTCCAGAC
pExKm5-*phiBp82.2△gp37-38*	pExKm5	*EcoRI, NotI*	*phiBP82.2_gp37* and *phiBP82.2_gp38*	Upstream_fw: ATTACCCTGTTATCCCTACCCGGGCCCGCAATGGCACGATGCGAG<br>Upstream_rev: TGTGCGCATTGCATTGACCACCATTCAAGTTGACAAGTGG<br>Downstream_fw: AATGGTGGTCAATGCAATGCGCACACGTGTAAC<br>Downstream_rev: CTCTAGGGATAACAGGGTAATCCCGGTGCCTTCGCAGAAAATCCG
pBIC, BBR1K::*phiBP82.2 _gp51*:: TsPurple chromoprotein gene	pBbR1k-GFP (addgene#35342)	*BamHI, NdeI*	*phiBP82.2_gp51*	forward: TTTTAAGAAGGAGATATACATATGTCAGCCGGCGGCGACCTTCG<br>reverse: ATGGGGCCCGCGAGTTGTGC
	pTsPurple chromoprotein (addgene#117848)		TsPurple gene	forward: GCACAACTCGCGGGCCCCATATGGCGAGCTTGGTTAAGAAAG<br>reverse: GAGATCCTTACTCGAGTTTGGATCCTTACGTCGCTTTTTCCGGC
